# Effects of Initial Performance, Gross Efficiency and 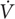
O_*2peak*_ Characteristics on Subsequent Adaptations to Endurance Training in Competitive Cyclists

**DOI:** 10.3389/fphys.2018.00713

**Published:** 2018-06-14

**Authors:** Knut Skovereng, Øystein Sylta, Espen Tønnessen, Daniel Hammarström, Jørgen Danielsen, Stephen Seiler, Bent R. Rønnestad, Øyvind Sandbakk

**Affiliations:** ^1^Centre for Elite Sports Research, Department of Neuroscience, Norwegian University of Science and Technology, Trondheim, Norway; ^2^Faculty of Health and Sport Sciences, University of Agder, Kristiansand, Norway; ^3^The Norwegian Olympic Federation, Oslo, Norway; ^4^Section for Sport Science, Lillehammer University College, Lillehammer, Norway

**Keywords:** cycling, performance, maximal oxygen consumption, gross efficiency, high intensity training, interval training

## Abstract

The present study investigated the effects of initial levels of cycling performance, peak oxygen uptake (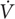
O_2peak_) and gross efficiency (GE) on the subsequent adaptations of these variables and their relationship following high-intensity training (HIT) designed to increase 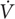
O_2peak_ in competitive cyclists. Sixty cyclists (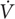
O_2peak_ = 61 ± 6 mL kg^-1^ min^-1^) were assigned a 12-week training program consisting of twenty-four supervised high-intensity interval training sessions and *ad libitum* low intensity training. GE was calculated at 125, 175, and 225 W and performance was determined by mean power during a 40-min time-trial (Power_40 min_). In addition to correlation analyses between initial level and pre- to post-intervention changes of the different variables, we compared these changes between four groups where participants were categorized with either low and/or high initial levels of 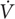
O_2peak_ and GE. Average volume of high- and low-intensity training during the 12-week intervention was 1.5 ± 0.3 and 8.3 ± 2.7 h·week^-1^, respectively. Following the 12-week training period, there was a significant increase in absolute and body mass normalized 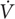
O_2peak_ and Power_40 min_ (*p* < 0.05) and a significant decrease in GE (*p* < 0.05) for all athletes pooled. There was no change in body mass following the 12-week training period. We found a moderate negative correlation between initial level of 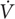
O_2peak_ and the change in 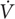
O_2peak_ following the training period (*r* = -0.32; *p* < 0.05). A small negative correlation was also found between initial Power_40 min_ and its change following training both when expressed in absolute power and power normalized for body mass (*r* = -0.27 and -0.28; both *p* < 0.05). A moderate negative correlation was also found between initial levels for GE and its change following training (*r* = -0.44; *p* < 0.01). There were no differences between the four groups based on initial levels of 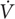
O_2peak_ and GE in the response to training on 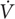
O_2peak_, GE, or Power_40 min_ (all *p* > 0.12). In conclusion, the present findings suggest that there are statistically significant effects of initial levels of cycling performance and 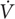
O_2peak_ and on the subsequent adaptations following a 12-week HIT program, but the small and moderate effects indicate limited influence on training practice.

## Introduction

Cycling performance requires high aerobic energy turnover and effective transfer of that energy to external power. Hence, the peak oxygen uptake (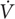
O_2peak_) and gross efficiency (GE), defined as the ratio of work rate to metabolic rate, are two key determinants of performance (Joyner and Coyle, 2008). Exercise training interventions focusing on high-intensity training (HIT) in cyclists have repeatedly shown enhanced 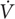
O_2peak_ as the main physiological adaptation (Lucia et al., 2000; Bacon et al., 2013) whereas GE has been reported to both be unaffected (Ronnestad et al., 2015) and improved (Hintzy et al., 2005; Hopker et al., 2009, 2010). However, the study of Ronnestad et al. (2015), where GE was unaffected, used an effort-based approach to control intensity, which likely induced higher intensity compared to the studies by Hopker et al. (2009, 2010) who regulated intensity by set blood lactate levels. The study by Hintzy et al. (2005) used a combination of moderate- and high-intensity, but the untrained participants in the study are a likely reason for the increased GE. Furthermore, sprint and strength training led to reduced oxygen cost of submaximal cycling (i.e., GE likely increased) (Paton and Hopkins, 2005), whereas low- and moderate-intensity training led to unchanged (Kristoffersen et al., 2014) or increased (Hopker et al., 2012) GE in cycling. Overall, the current literature indicates that both training intensity and fitness level influence the responses on GE.

It is generally believed that greater training loads, achieved through both volume of training and sufficient intensity, are required to trigger 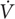
O_2peak_ or performance adaptations in individuals with high compared to low initial levels of 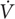
O_2peak_ or performance. In previous studies, there has been reported an effect of initial level of 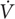
O_2peak_ on the response to exercise (Saltin et al., 1969), but more recently no significant correlation between initial 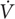
O_2peak_ and the response to a training intervention was reported (Kohrt et al., 1991; Skinner et al., 2001). However, these studies (Kohrt et al., 1991; Skinner et al., 2001) do not include any measures of performance, and the participants had a relatively low initial level of fitness (reported 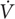
O_2peak_ < 31.8 mL kg^-1^ min^-1^). As such, inference to performance and/or more highly trained populations cannot be made based on these studies.

The interaction between 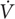
O_2peak_ and GE adaptations in response to a training intervention requires further elucidation. An inverse relationship between 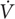
O_2peak_ and GE has been found (Lucia et al., 2002) and also an inverse relationship between the change in GE and 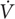
O_2peak_ following a training intervention (Santalla et al., 2009; Hopker et al., 2012). However, none of these interventions led to increased 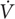
O_2peak_ at the group level.

Therefore, the purpose of the present study was to investigate how baseline characteristics of cycling performance, 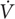
O_2peak_ and GE influence subsequent adaptations of these parameters and their interplay following a 12-week high-intensity intervention designed to increase 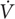
O_2peak_ in competitive cyclists. We hypothesized that a large training load would lead to increased 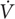
O_2peak_ and reduced GE, but there would be no influence of initial levels of 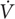
O_2peak_ and GE.

## Materials and Methods

Sixty-three male competitive cyclists (38 ± 8 year, 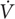
O_2peak_: 61 ± 6 mL kg^-1^ min^-1^) were recruited to take part in the current multicentre study, involving three test centers completing the same experimental trial. All participants were categorized as well-trained (Jeukendrup et al., 2000) with 9 ± 3 h of weekly training in the year prior to participation. All participants completed the intervention, however, three participants were excluded from the final analyses due to absence from post-testing. The study was approved by the ethics committee of the Faculty for Health and Sport Science, University of Agder, and registered with the Norwegian Social Science Data Services (NSD). All athletes gave their verbal and written informed consent prior to study participation. The present study is part of a larger research project and thus, the intervention period, testing procedures and instrument are described in brief. A detailed description of the intervention period, testing procedures, and instruments can be found in Sylta et al. (2016).

### Intervention Period

In brief, after a 6-week preparation and familiarization period, the training intervention consisted of three 4-week mesocycles. During the last week in each mesocycle, participants were advised to reduce low-intensity training (LIT) volume by 50% compared to previous weeks. In addition, HIT session frequency was reduced from 3 to 1 during the last week of each mesocycle. In total, each participant was prescribed twenty-four supervised HIT sessions in addition to testing and self-organized *ad libitum* LIT. All training was recorded using an online training diary and a heart rate monitor was worn for all exercise training. Three different HIT session models were utilized and all included a self-selected warm up of 20–30 min of LIT, followed by four high intensity interval efforts of 4, 8, or 16 min at a self-selected cadence, separated by 2 min rest, followed by 10–20 min of LIT as a cool-down. All HIT training was performed while supervised on Computrainer cycling trainers (RacerMate Inc., Seattle, WA, United States) using the participants’ own bikes and, additionally, blood lactate measurements were taken from a selection of the participants on each session. The participants were instructed to cycle at their maximal sustainable intensity for the entire session, and provided with continuous feedback regarding cadence, heart rate (HR), and power output. All participants were prescribed the same number of the three different HIT session models.

### Testing Procedures

In brief, on test *day 1*, a submaximal, incremental exercise test consisting of 5-min steps was performed on a bicycle ergometer at work rates of 125, 175, and 225 W. 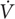
O_2_ and the respiratory exchange ratio (RER) was used to calculate the metabolic rate during the three work rates. The work rate was then divided by the metabolic rate to calculate GE. The 125, 175, and 225 W work rates used to calculate GE corresponded to 34 ± 4, 48 ± 5, and 61 ± 6% of the participants’ peak power output (PPO) and to 43 ± 4, 53 ± 5, and 65 ± 6% of their 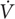
O_2peak_ achieved during the incremental test. 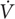
O_2_, RER, and HR were measured during the last 2.5 min of each step when a steady state condition had occurred. Blood lactate was measured after 4.5 min of each step. After 10 min recovery, an incremental test to exhaustion was performed starting with 1 min of cycling at 3 W kg^-1^ (rounded down to nearest 50 W) and subsequent increases of 25 W every minute. Strong verbal encouragement was provided throughout the test. 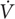
O_2peak_ was calculated as the average of the two highest consecutive 30-s 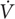
O_2_ measurements and PPO was calculated as the mean power output during the final 60 s the participants were able to maintain power output during the incremental test. HR_peak_ was observed during the final 5 s before exhaustion and blood lactate was measured 60 s post-exhaustion.

On test day 2, participants performed a 40-min time trial (Power_40min_) after a 30-min warm-up at a self-selected power output. The Power_40min_ test was conducted under supervision in a well-ventilated room. The temperature and relative humidity were similar at the pre- and post-tests and on both occasions *ad libitum* water intake was allowed. Participants were blinded to all feedback except for elapsed time and the participants were instructed to cycle at the highest possible mean power output for 40 min.

### Instruments and Materials

In brief, all physiological tests were performed on a cycling ergometer [Velotron (RacerMate, Seattle, WA, United States) or Lode Excalibur Sport (Lode B. V., Groningen, Netherlands)] adjusted to the participant’s preference. The type of ergometer was consistent at pre- and post-tests. Participants were instructed to remain seated during all tests, with self-selected cadence. Performance tests and all HIT sessions were performed using each participant’s personal road bike mounted on Computrainer Lab^TM^ trainers (RacerMate, Seattle, WA, United States), calibrated according to the manufacturer’s specifications. 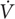
O_2_ was measured using Oxycon Pro^TM^ with mixing chamber (Oxycon, Jaeger GmbH, Hoechberg, Germany) calibrated using gases of known concentrations before every test. The flow turbine (Triple V, Erich Jaeger) was calibrated using a 3L calibration syringe (5530 series; Hans Rudolph, Kansas, MO, United States). HR was measured using Polar V800 (Polar Electro Oy, Kempele, Finland) and blood lactate was measured using capillary blood samples taken from the fingertip (Biosen C-Sport, EKF diagnostics, Cardiff, United Kingdom).

### Data Analysis

**FIGURE 1:**
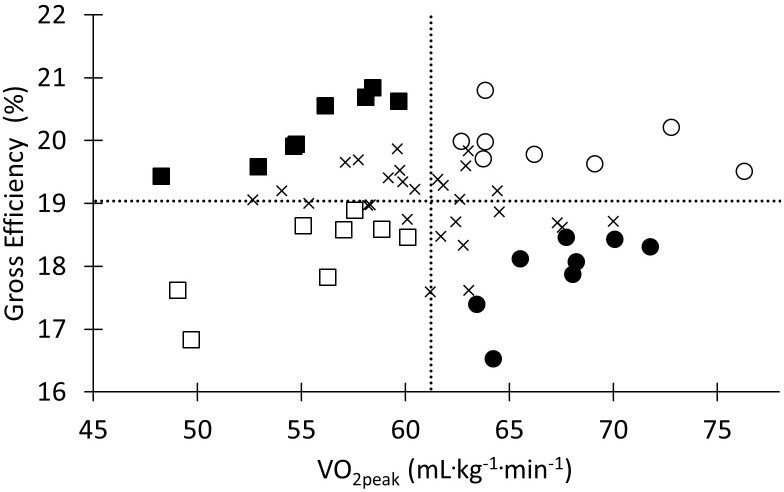
Participant distribution of 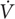
O_2peak_ relative to body mass and GE at the baseline measurement. Open circles indicate participants in the HH (high 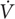
O_2peak_ and high efficiency) group. Filled circles indicate participants in the HL (high 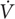
O_2peak_ and low efficiency) group. Filled squares indicate participants in the LH (low 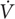
O_2peak_ and high efficiency) group. Open squares indicate participants in the LL (low 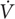
O_2peak_ and low efficiency) group.X indicates subjects not included in any of the sub groups (*N* = 28). Dotted lines indicate the mean value for 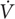
O_2peak_ and gross efficiency.

Four subgroups (each with *N* = 8) were identified from the complete cohort, characterized by high or low 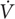
O_2peak_ relative to body mass and high or low GE. 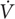
O_2peak_ and GE rank within the cohort from the pre-test was used as criteria for selecting participants to the respective groups (**Figure 1**). The HH (high GE and high 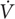
O_2peak_) and LL (low GE and low 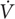
O_2peak_) groups were selected to yield the highest and lowest average rank for 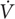
O_2peak_ and GE, respectively. The HL (high GE and low 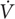
O_2peak_) group was selected to have the highest 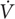
O_2peak_ rank while maintaining an average rank as close to the mean as possible and LH (low GE and high 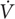
O_2peak_) group was selected to have the highest possible GE score while maintaining an average rank as close to the mean as possible.

### Statistical Analyses

Pearsons correlation coefficients were calculated to determine relationships between cycling performance, 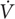
O_2peak_ and GE and a descriptors of effect sizes of the correlations were calculated according to http://www.sportsci.org/resource/stats/effectmag.html. Differences between PRE and POST conditions were evaluated using a one-way ANOVA. Differences in responses among the four different groups were evaluated using a two-way repeated measures ANOVA. We calculated the smallest worthwhile change in performance as 0.20 multiplied with the standard deviation (SD) at the pre-test (Hopkins et al., 2009). All data analyses were conducted using SPSS 22.0 (SPSS Inc, Chicago, IL, United States) and are presented as mean ± SD with statistical significance accepted as α ≤ 0.05.

## Results


**FIGURE 2:**
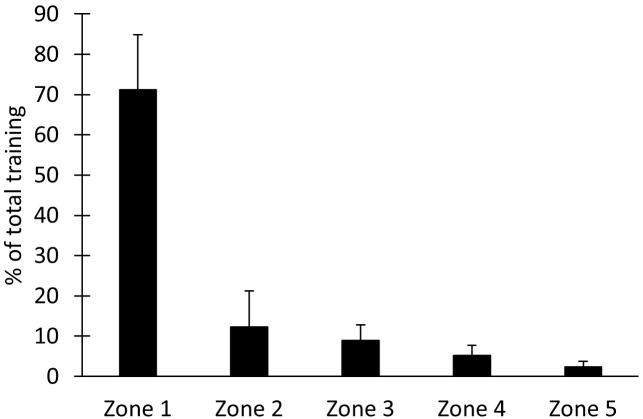
Intensity distribution from heart rate monitored training throughout the study. Zone 1: <75% of HR_peak_, zone 2: 75–85% HR_peak_, zone 3: 85–90% of HR_peak_, zone 4: 90–95% of HR_peak_, and zone 5: >95% of HR_peak_.

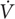
O_2peak_ ranged from 48 to 76 mL·kg^-1^ min^-1^ and GE from 16.5 to 20.8%. The average Power_40min_ ranged from 194 to 342 W and 2.3 to 4.6 W kg^-1^ when expressed as absolute values and normalized to body mass, respectively. A plateau in 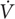
O_2_ occurred in 54 and 58 out of the 60 pre- and post-tests, respectively. Following the 12-week training intervention, there was a significant increase in 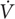
O_2peak_, PPO, and Power_40min_ but there was a decrease in GE (**Table 1**; all *p* < 0.05). The weekly training volume during the intervention was 10 ± 3 h, of which 97 ± 4% was endurance training. The mean power output during all HIT intervals was 310 ± 40 W and average blood lactate taken during the sessions was 8.7 ± 4.0 mmol L^-1^. The overall intensity distribution for all training based on heart rate data is presented in **Figure 2**.

**Table 1:** Pre- and post-test values of performance and physiological factors following the 12-week high-intensity training intervention in 60 well-trained cyclists.

	Pre	Post
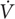 O_2peak_ (mL min^-1^)	4859 ± 462	5078 ± 484*
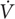 O_2peak_ (mL kg^-1^ min^-1^)	61 ± 6	65 ± 6*
GE (%)	19.0 ± 0.9	18.6 ± 0.9*
PPO (W)	372 ± 40	384 ± 29*
PPO (W kg^-1^)	4.7 ± 0.5	4.9 ± 0.4*
Power_40min_ (W)	282 ± 30	301 ± 31*
Power_40min_ (W kg^-1^)	3.6 ± 0.4	3.9 ± 0.4*
Peak blood lactate (mmol L^-1^)	12.1 ± 2.3	12.7 ± 2.1
RER	1.15 ± 0.1	1.15 ± 0.1
Body mass (kg)	80 ± 8	78 ± 8

*Peak oxygen uptake (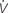
O_2peak_), gross efficiency (GE) averaged from 125, 175, and 225 W, peak power output from the incremental test (PPO), average power output during the 40-min time trial (Power_40min_), peak blood lactate, and RER-values from the incremental test and body mass are presented as arithmetic mean (±SD). Asterisks indicate a significant change from the pre- to post-test (p < 0.05)*.

### Initial Characteristics

**FIGURE 3:**
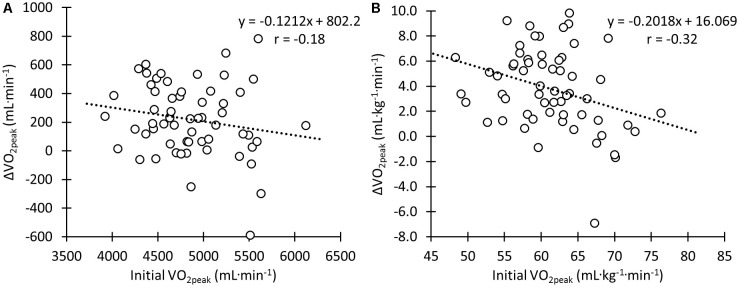
Correlation between the change in 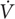
O_2peak_ during the training intervention and initial 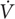
O_2peak_ in absolute values **(A)** and normalized for body mass **(B)**.

Initial absolute 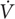
O_2peak_ did not correlate significantly with the change in 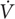
O_2peak_ (**Figure 3A**) following the 12-week intervention (**Figure 2A**; *r* = -0.18; *p* = 0.17). However, for 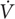
O_2peak_ expressed relative to body mass, there was a moderate significant negative correlation between the initial values and the change in 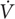
O_2peak_ following the intervention (**Figure 3B**; *r* = -0.32; *p* < 0.05). Smallest worthwhile changes for body mass normalized 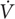
O_2peak_, GE, and body mass normalized performance during the Power_40min_ were 1.2 mL kg^-1^ min^-1^, 0.18%, and 0.08 W kg^-1^, respectively.

**FIGURE 4:**
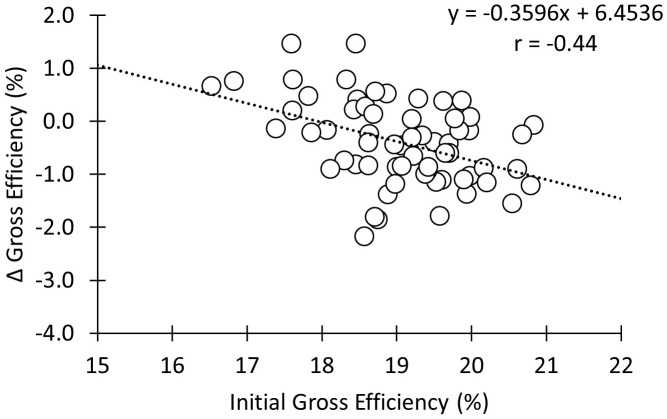
Correlation between the percentage change in gross efficiency during the training intervention and initial gross efficiency.

There was a significant negative moderate correlation between initial GE and percentage change in GE following the 12-week intervention (**Figure 4**; *r* = -0.44; *p* < 0.01). There was no change in cadence during the stages used for GE calculation from the pre- to the post-test (*p* = 0.35).

**FIGURE 5:**
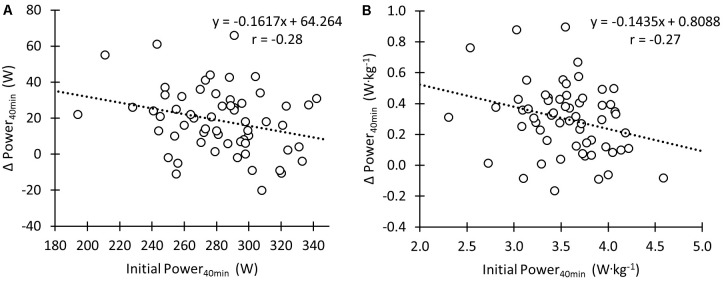
Correlation between the change in time trial performance during the training intervention and initial time trial performance (Power_40min_) in absolute units **(A)** and normalized for body mass **(B)**.

Initial performance during the Power_40min_ displayed a small negative correlation with the change in performance following the training intervention both when expressed as absolute values (**Figure 5A**; *r* = -0.28; *p* < 0.05) and relative to body mass (**Figure 5B**; *r* = -0.27; *p* < 0.05). Initial PPO showed small and moderate correlations with the change following the intervention both for absolute power output (*r* = -0.23; *p* = 0.08) and values relative to body mass (*r* = -0.42; *p* < 0.01), respectively.

### GE and 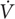
O_2peak_ Relationship and Their Relationship With Performance

**FIGURE 6:**
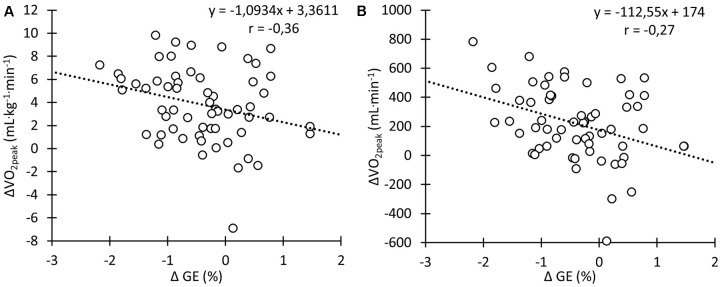
Correlation between changes in 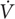
O_2peak_ relative to body mass **(A)** and absolute 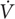
O_2peak_ **(B)** and changes in gross efficiency (GE) during the training intervention.

There was a moderate negative correlation between GE and both absolute 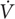
O_2peak_ (**Figure 6A**; *r* = -0.36, *p* < 0.01) and 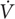
O_2peak_ relative to body mass (**Figure 6B**; *r* = -0.27, *p* < 0.05).

**FIGURE 7:**
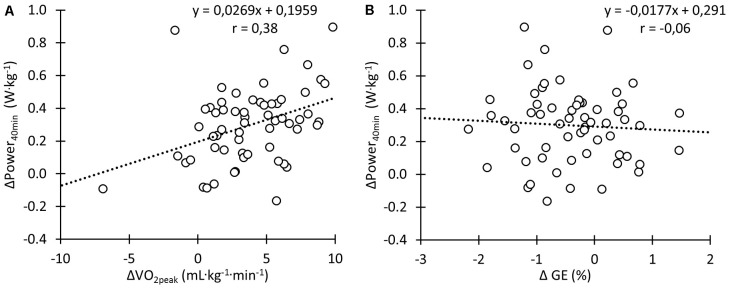
Correlation between change in 40-min time trial performance normalized for body mass and change in 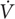
O_2peak_ normalized for body mass **(A)** and between changes in 40-min time trial performance normalized for body mass and changes in gross efficiency **(B)** during the training intervention.

There were significant moderate correlations between change in performance during the Power_40min_ and change in absolute 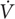
O_2peak_ (*r* = 0.30, *p* < 0.05) and 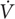
O_2peak_ relative to body mass (**Figure 7A**; *r* = 0.38, *p* < 0.01). There was no significant correlation between change in performance during the Power_40min_ and change in GE (**Figure 7B**; *r* = -0.06, *p* = 0.63).

### Sub-group Analyses

Characteristics of the participants included in the four sub-groups are presented in **Table 2**. The HH group showed the best performance on both the incremental and the time-trial test, but only when the results where normalized for body mass. Total training volume, LIT, and HIT did not differ between the four subgroups (all *p* > 0.36).

**Table 2:** Pre-test characteristics and training data during the 12-week intervention period for the four sub-groups.

	Hi 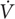 O_2peak_/Hi GE	Hi 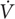 O_2peak_/Low GE	Low 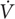 O_2peak_/Hi GE	Low 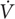 O_2peak_/Low GE
Age (years)	32 ± 6^cd^	36 ± 6^d^	41 ± 6^a^	44 ± 4^ab^
Height (cm)	181 ± 9	184 ± 5	181 ± 4	184 ± 6
Heart rate peak (bpm)	194 ± 7	194 ± 10	184 ± 9	185 ± 13
Body mass (kg)	71.3 ± 7.9^bcd^	79.6 ± 2.7^ad^	80.9 ± 3.2^ad^	88.9 ± 10.3^abc^
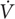 O_2peak_ (mL min^-1^)	4784 ± 509^b^	5362 ± 268^acd^	4622 ± 460^b^	4916 ± 488^b^
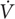 O_2peak_ (mL kg^-1^ min^-1^)	67.3 ± 5.0^cd^	67.4 ± 2.8^cd^	57.2 ± 3.4^ab^	55.5 ± 4.1^ab^
GE (%)	19.9 ± 0.4^bd^	17.9 ± 0.7^ac^	20.1 ± 0.7^bd^	18.2 ± 0.7^ac^
PPO (W)	367 ± 43	394 ± 38	367 ± 43	361 ± 46
PPO (W kg^-1^)	5.2 ± 0.4^cd^	4.9 ± 0.4^cd^	4.5 ± 0.5^abd^	4.1 ± 0.4^abc^
Power_40min_ (W)	283 ± 22	282 ± 20	274 ± 40	281 ± 39
Power_40min_ (W kg^-1^)	4.0 ± 0.3^abc^	3.5 ± 0.2^a^	3.4 ± 0.5^a^	3.2 ± 0.5^a^
HIT volume (h week^-1^)	1.4 ± 0.3	1.5 ± 0.3	1.5 ± 0.3	1.6 ± 0.7
LIT volume (h week^-1^)	9.1 ± 3.7	7.3 ± 2.0	7.3 ± 2.4	7.7 ± 2.3

*Pre-test values for the HH (high 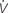
O_2peak_ and high efficiency), HL (high 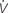
O_2peak_ and low efficiency), LH (low 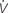
O_2peak_ and high efficiency), and LL (low 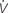
O_ 2peak_ and low efficiency) groups. 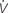
O_2peak_, gross efficiency (GE), peak power output from the incremental test (PPO) and average power output during the 40-min time trial (Power_40min_) are presented as mean (±SD). ^a^Indicates a difference from HH (p < 0.05). ^b^Indicates a difference from HL (p < 0.05). ^c^Indicates a difference from LH (p < 0.05). ^d^Indicates a difference from LL (p < 0.05)*.

**FIGURE 8:**
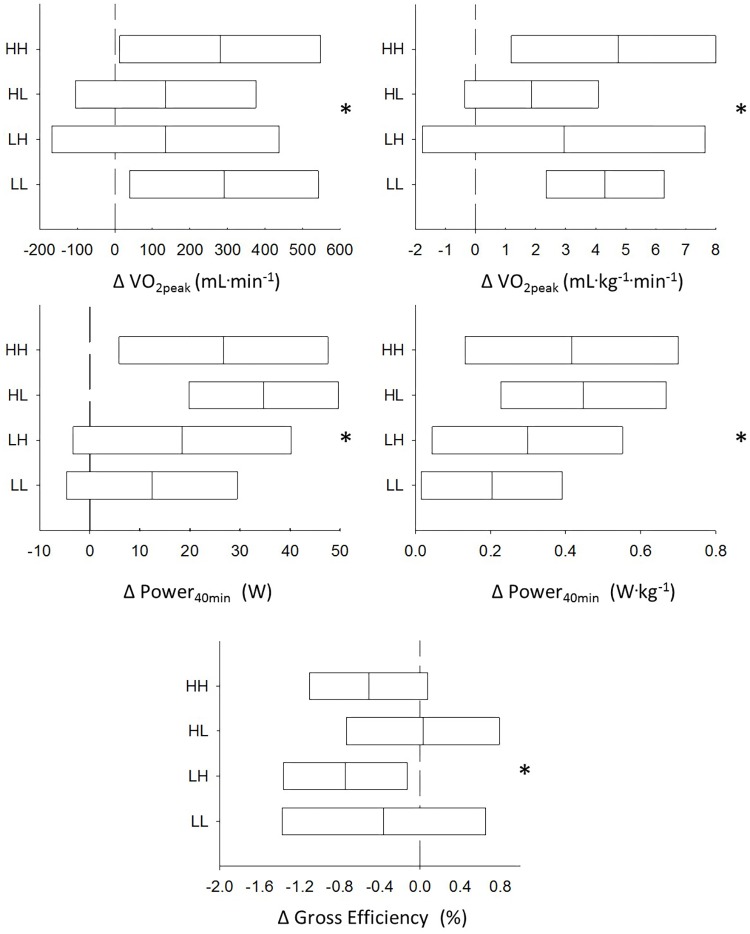
Group responses from the pre-test to the post-test for the HH (high 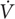
O_2peak_ and high efficiency), HL (high 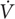
O_2peak_ and low efficiency), LH (low 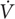
O_2peak_ and high efficiency), and the LL (low 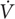
O_2peak_ and low efficiency) groups for 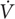
O_2peak_, time trial performance and gross efficiency (GE). ^∗^Indicates a significant main effect of the training intervention.

There was a significant main effect of the training intervention on 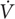
O_2peak_, Power_40min_ (**Figure 8**; all *p* < 0.05) and PPO (not shown) during the incremental test. However, there was no difference between the four groups in the change in 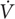
O_2peak_, PPO, Power_40min_ or GE (all *p* > 0.12) and all four groups maintained the selection characteristics following the intervention (i.e., the HH and HL had higher 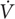
O_2peak_ compared to the LH and LL and the HH and LH had higher GE compared to the HL and LL) (all *p* < 0.05).

## Discussion

The purpose of the present study was to investigate how the initial characteristics of cycling performance, 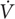
O_2peak_, and GE, as well as their interplay, influences subsequent adaptations in these parameters following a 12-week HIT intervention in well-trained cyclists.

The training intervention led to an increase in performance and 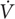
O_2peak_ but a decrease in GE. Furthermore, we found significant associations between the athletes’ initial levels of GE, 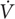
O_2peak_, and performance and subsequent adaptations of that same parameter. However, parameter specific initial levels explained only 4–18% of the variance in adaptations, and when we compared our four groups with combinations of high and/or low initial levels of GE and 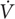
O_2peak_, no differences in adaptations were found among groups. Overall, our study shows that the impact of initial characteristics of cycling performance, 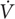
O_2peak_, and GE on the subsequent responses to these parameters are relatively small, and the effect disappears if we investigate the relationship in groups based on initial GE and 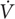
O_2peak_ characteristics in combination.

While the present study shows significant effects of initial performance and 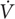
O_2peak_ on the subsequent adaptation to HIT in highly trained cyclists, the effect is only moderate. An effect of initial fitness on the 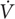
O_2peak_ responses has also been reported previously in less trained individuals (Saltin et al., 1969), although other studies (Kohrt et al., 1991; Skinner et al., 2001) showed no effect. The HIT element in the present study had an overall positive effect on 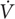
O_2peak_ and endurance performance, which differs from the previous studies that utilized a lower training intensity (e.g., 75% of 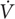
O_2peak_ (Skinner et al., 2001)). Furthermore, the total training load added in our study seemed to be sufficient to elicit an overload stimulus and thereby trigger endurance adaptions in most of the athletes. This is exemplified by a recent study where further increasing the training load effectively elicited training adaptations in participants who were unresponsive to 180 min of moderate to high intensity exercise per week (Montero and Lundby, 2017). However, even in our study, the observed improvements were relatively modest, which may partly be due to differences in how optimal the added training load was for each individual participant. In addition, the subsequent recovery phase is of importance for adaptations, and e.g., subjects need to be healthy and avoid other factors which might negatively influence adaptations.

In contrast to the positive effects of the 12-week high intensity training intervention on 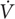
O_2peak_ (∼5%), GE declined slightly following the intervention (∼2%), and there was a moderate association between initial level of GE and the subsequent response. However, there were no significant differences in adaptation between the groups with high and low GE. Previous literature examining changes in GE following high intensity training reported no effect (Ronnestad et al., 2015) or an increase in GE when utilizing an exercise intensity equivalent to five heart rate beats above the work rate eliciting 4 mmol⋅L^-1^ of blood lactate (Hopker et al., 2010). However, previous studies in participants of a similar performance level to our participants, who utilized low- to moderate-intensity training, reported improved GE and a significant positive relationship between changes in GE and performance (Hopker et al., 2010, 2012).

A potential explanation for the different effect of training on GE between our study and others might be the HIT applied in our study, which primarily influenced performance via enhanced 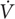
O_2peak_ (Sylta et al., 2016)). Previous studies (Hopker et al., 2010, 2012) have used a lower intensity compared to the present study, and a lower intensity may be more effective for enhancing GE at a submaximal work rate. In the present study, training was executed as HIT with the addition of *ad libitum* LIT. A possible influencing factor on the decline in GE in the present study may be the low amount of training as moderate-intensity exercise, which may be important for maintaining GE (Hopker et al., 2009; Kristoffersen et al., 2014).

Intensity in the present study was controlled utilizing an effort based approach where the participants are instructed to aim to achieve the highest possible average power output within each session (Seiler et al., 2013). This is the same approach used by Ronnestad et al. (2015) who demonstrated unchanged GE after HIT training. It is possible that the effort based intensity control leads to higher intensity, as shown through the high blood lactate levels during intervals in the present (i.e., average blood lactate of 8.9 mmol L^-1^) and a previous study using intervals of 4 to 8 min duration (Seiler et al., 2013), compared to the approaches used by Hopker et al. (2010) who demonstrated increased GE with intensity based on absolute blood lactate levels of 4 mmol L^-1^ + 5 heart rate beats per minute. Furthermore, Hopker et al. (2009, 2010) proposed that training at moderate intensity, eliciting less than 4 mmol L^-1^ blood lactate, is important for maintaining GE. Hence, training at (very) high-intensity may lead to unchanged or declined GE, whereas training at lower intensity may have the opposite effect.

However, the decline in GE may also be influenced by the fact that it was calculated at a moderate intensity (i.e., the average of 125, 175, and 225 W) and might not reflect what occurs at higher workloads (i.e., training intensity average interval work rate of 310 W). Additionally, since 225 W exceed 60% of PPO, there is a possibility that the 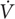
O_2_ slow component influence our measurements slightly, which could have led to an overestimation of the decline in GE from pre- to post-test. However, since there was no difference in the change in GE for the three work rates used for GE calculation and the corresponding RER measurements were below 1.0, a possible influence would be minor.

As expected, we found a positive relationship between change in cycling performance and change in 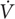
O_2peak_. In contrast, change in GE was not related to a change in performance. This inverse relationship between the change in GE and 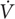
O_2peak_ (**Figure 5**) corresponds with previous findings (Lucia et al., 2002; Hopker et al., 2012). However, in contrast to previous studies which have shown an increase in GE and small changes in 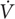
O_2peak_ (Lucia et al., 2002; Hopker et al., 2012), we show the same inverse relationship when the average GE decreases and the average 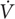
O_2peak_ increases.

Also contrary to previous findings (Hopker et al., 2012) is an increased performance despite a decrease in GE. Although the 12-week HIT intervention led to a large increase in 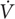
O_2peak_ that positively influenced cycling performance, it appears that cycling efficiency is slightly reduced, especially in those with large 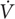
O_2peak_ improvements. However, it is important to keep in mind the relatively short duration of the intervention executed in this study. As demonstrated by the findings in this study, the decrease in GE over a relatively short period where the objective of training is to increase 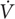
O_2peak_ was not detrimental to performance due to the improved 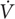
O_2peak_. However, in general, long-term decreases in GE can be detrimental to performance. Since studies using low- and moderate-intensity training, as well as sprint and strength training (Paton and Hopkins, 2005; Hopker et al., 2012) have shown increases in GE, cyclists should likely use a combination of different training intensities to optimize their long-term performance development. This also represents a typical training pattern over a season for elite cyclists, where a combination of intensities and periods with different focus is employed (Lucia et al., 2000; Hopker et al., 2009). Although different types of training influence GE and 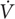
O_2peak_ differently, both high and moderate intensity training may be necessary for optimal performance increases during a competitive season. Additionally, the long-term effect on GE and 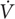
O_2peak_ is potentially different from the effects seen in this relatively short 12-week intervention compared to the effect on performance.

We believe the finding of a small effect of baseline level has important practical implications and demonstrates that if an appropriate training stimulus is administered, the initial physiological characteristics have little effect on the training adaptation in a large cohort of well-trained athletes. It demonstrates that an appropriate training stimulus can further enhance performance, independent of the initial physiological characteristics of even well-trained athletes. A known limitation of the present arises from the baseline characteristic and change following the intervention are not independent measurements, we violate the assumption of independence in the correlation analyses. This may lead to an effect known as regression to the mean (Altman and Bland, 1994) which ultimately would lead to an overestimation of the correlation. When calculated using Oldham’s correction to minimize the effect, the correlations in the present study were weakened. The additional finding that the groups based on differences in baseline characteristics show no difference in the adaptation to the training intervention also supports our interpretation. Although the groups in the present study where comprised of only eight athletes which limit statistical power with multiple comparisons, however, the group size is comparable to similar studies on training adaptation.

## Conclusion

In conclusion, the present study demonstrates statistically significant, but practically trivial effects of initial levels of 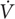
O_2peak_ and performance on subsequent adaptations following a 12-week HIT intervention in well-trained cyclists. In contrast to the improvements in performance and 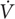
O_2peak_ following this intervention, GE was reduced and the changes in GE negatively correlated with changes in 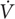
O_2peak_. However, when comparing adaptations between groups with different levels of 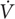
O_2peak_ and/or GE, we found no differences. Overall, this study indicates that the effects of initial level of performance and physiological capacities on subsequent adaptations are relatively small, and the effect disappears if we investigate the relationship in groups based on GE and 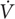
O_2peak_ characteristics.

## Author Contributions

KS, ØSa, ET, JD, BR, and ØSy contributed in conceptualization the study. KS, ØSa, DH, and JD contributed in data collection. KS, ØSa, DH, and ØSy contributed in data handling and statistical analysis. KS, ØSa, ET, DH, JD, SS, BR, and ØSy contributed in preparing the manuscript.

## Conflict of Interest Statement

The authors declare that the research was conducted in the absence of any commercial or financial relationships that could be construed as a potential conflict of interest.
